# Design and first-round commissioning result of the SASE beamline at the Shanghai Soft X-ray FEL facility

**DOI:** 10.1107/S1600577523009438

**Published:** 2024-01-01

**Authors:** Chaofan Xue, Zhi Guo, Haigang Liu, Jiahua Chen, Yajun Tong, Jiadong Fan, Huaidong Jiang, Zhi Liu, Ximing Zhang, Renzhong Tai

**Affiliations:** a Shanghai Advanced Research Institute, Chinese Academy of Sciences, Shanghai 201210, People’s Republic of China; b Shanghai Synchrotron Radiation Facility, Shanghai 201204, People’s Republic of China; c ShanghaiTech University, Shanghai 201210, People’s Republic of China; Advanced Photon Source, USA

**Keywords:** free-electron laser, SASE beamline, online spectrometer, focusing spot

## Abstract

The Shanghai Soft X-ray Free-Electron Laser (SXFEL) is the first X-ray free-electron laser facility in China. The SASE beamline, which consists of a pink-beam branch and a mono-beam branch, is one of the two beamlines in the Phase-I construction. The pink-beam branch opened for users in 2023 after successful first-round beamline commissioning. In this paper, the design of the beamline is presented and the performance of the pink-beam branch is reported. The measured energy-resolving power of the online spectrometer is over 6000 @ 400 eV. The focusing spot size of the pink beam is less than 3 µm in both the horizontal and vertical at the endstation.

## Abstract 2

The design and first-round commissioning results of the SASE beamline at the Shanghai Soft X-ray FEL facility are presented.

## 1. Introduction

Owing to their many unique properties, such as transversely coherent and ultrashort-pulsed sources, high brightness and peak power, tunable wavelengths and wide spectral ranges (Saldin *et al.*, 1995 ▸; Tiedtke *et al.*, 2004 ▸; Amann *et al.*, 2012 ▸; Togashi *et al.*, 2013 ▸; McNeil & Thompson, 2010 ▸), electron linac-based free-electron laser (FEL) sources have been widely applied in numerous basic scientific research fields – for example, condensed matter physics, advanced materials and surface physics, atomic and molecular physics, chemistry and biology (Mankowsky *et al.*, 2014 ▸; Beaud *et al.*, 2014 ▸; Rohringer & Santra, 2007 ▸; Wernet *et al.*, 2015 ▸; Liu *et al.*, 2013 ▸). Up to now, several FEL facilities (Ackermann *et al.*, 2007 ▸; Emma *et al.*, 2010 ▸; Ishikawa *et al.*, 2012 ▸; Allaria *et al.*, 2012 ▸; Kang *et al.*, 2017 ▸; Prat *et al.*, 2020 ▸; Decking *et al.*, 2020 ▸), from low repetition rate to high repetition rate, have been built or are under construction worldwide to meet the needs of scientists for ultra-high-brightness and ultra-short-pulse sources.

As the first X-ray FEL facility in China, the Shanghai Soft X-ray Free-Electron Laser (SXFEL) (Zhao *et al.*, 2017 ▸; Liu *et al.*, 2022 ▸) consists of one linac, two undulator lines, two beamlines and five experimental endstations focusing on dynamic and radiation-damage-free imaging, ultrafast physical phenomena, surface and ultrafast chemical processes, and atomic and molecular physics. The linac can provide a high-quality electron beam with energy of 1.5 GeV, charge of 0.5 nC, peak current of about 700 A and normalized project emittance of about 1.5 mm mrad. The two undulator lines share the electron bunch from the linac and are operated under different operating modes – self-amplified spontaneous emission (SASE) mode and external seed mode – to produce FEL radiation with 100 fs (FWHM) pulse length at 50 Hz repetition rate. The SASE beamline, which covers the wavelength from 1.2 nm to 12 nm, mainly consists of three parts: front-end area, photon transport system and photon beam diagnostic system. The front-end area is designed for equipment and personal safety protection. The photon transport system will deliver FEL pulses produced by the upstream undulators to different experimental endstations including monochromatizing the pink beam, distributing FEL pulses to different branches and focusing the photon beam to the sample. The photon beam diagnostic system is applied to measuring the basic properties of the beam like spectral structure and pulse energy besides the pulse profile monitoring.

In this paper, the design of the SASE beamline at SXFEL is presented and the initial commissioning results of the SASE beamline are also reported.

## 2. Beamline layout

**Figure 1:**
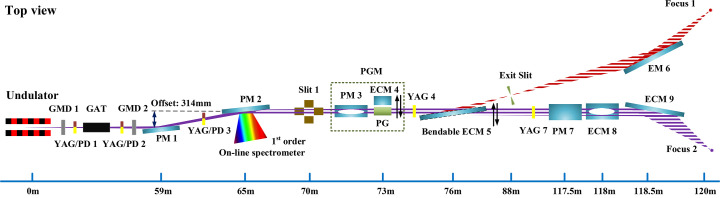
Layout of the SASE beamline at SXFEL.

The optical layout of the SASE beamline is shown in Fig. 1 ▸. The first optics are offset mirrors which consist of two plane mirrors located at 59 m and 65 m downstream of the source. An offset of 314 mm is generated by the offset mirrors in the horizontal to block the upstream high-energy radiation. For the offset mirrors, the grazing incident angle is the same for both mirrors, *i.e.* 1.5°, to keep the outgoing beam parallel to the incoming beam. In the central area of the second plane mirror a small grating is ruled to disperse the incoming beam, and the dispersed beam is recorded by a CCD camera. In this way the spectrum of the FEL pulse can be measured shot by shot, which is quite important for both accelerator and endstation in order to know the spectral structure of a SASE pulse. The area of the grating is 80 mm × 5 mm, which only covers a small portion of the reflected beam (∼3%), while the other part is deflected into the zero order and straight forward towards the beamline, and the wavefront can be maintained as far as possible. The grating is chosen to be a variable-line-spacing (VLS) type to focus the dispersive beam onto the CCD camera and minimize the aberration as a result of the optimization of the VLS parameters. A four-jaw slit is located at 70 m from the source point to define the acceptance angle of the downstream optics in the beamline. A variable-included-angle VLS plane-grating monochromator (PGM) is located at 73 m from the source point to select photons in a very narrow bandwidth. A 400 lines mm^−1^ grating is designed in the PGM to balance energy resolution and pulse length since the pulse will be stretched after passing through the grating. A FEL pulse with high energy resolution or high peak intensity is required according to different experimental methods and scientific objectives. In order to meet different needs, the plane mirror in the PGM can be moved out of the beam path to allow the pink beam to go through the vacuum chamber without passing through the grating. The beamline is separated into two branches after the monochromator. The FEL pulses can be distributed in the horizontal to a different branch by moving ECM5 (elliptical cylindrical mirror 5), located 76 m from the source point, in or out of the beam path. When ECM5 is moved out of the beam path, the photon beam directly reaches the Kirkpatrick–Baez (KB) focusing mirrors after passing through the offset mirrors. The KB system includes three mirrors: besides the two elliptical cylindrical mirrors which focus the photon beam in the horizontal and vertical, a plane mirror is added as the first mirror to guarantee that the photon beam is incident to the experimental endstation horizontally. The spacing between mirrors is 0.5 m in the focusing system and the last focusing mirror is located at 118.5 m from the source point. The first sample point is located at 1.5 m downstream of the last focusing mirror. Since only five mirrors are passed through, the wavefront can be well preserved. Therefore, this focus is suitable for experiments requiring high peak intensity or ultrashort pulse length such as coherent diffraction imaging, *etc*. When ECM5 is moved into the beam path, the photon beam is deflected to another branch. In most cases this branch is designed to transport monochromatic beam. The imaging distance of the grating is 15 m, whereupon an exit slit is used to selected photons of the required wavelength. The position of the exit slit is also the focus of ECM5. Consequently, the exit slit also acts as the secondary source point for the downstream optics. If ultrashort pulse length but not high energy-resolving power is required in this branch, the grating in the PGM will be replaced by an elliptical focusing mirror whose imaging distance is the same as that of the grating. An ellipsoidal mirror EM 6 located at 118.5 m is applied to focus the beam in both the horizontal and vertical simultaneously to 120 m. Based on the PGM with high energy-resolving power, this focus is suitable for carrying out spectroscopy related experiments. All of the optics in the SASE beamline are coated with B_4_C owing to its high single pulse damage threshold and high reflectivity in the optimized energy range. The basic parameters of the optics in the SASE beamline are summarized in Table 1 ▸.

A gas attenuator located in front of PM1 is applied to adjust the pulse energy to protect the optics or other equipment in the beamline from being damaged by a high-energy pulse in the commissioning stage or experiment preparation. The effective absorption length of the gas attenuator is 6 m with argon and nitro­gen as working gas. The maximum operating pressure is 0.8 Torr to provide up to four orders of magnitude of attenuation. Two gas monitors are installed in front of and behind the gas attenuator to monitor the pulse energy shot by shot. Both gas monitors are installed in the differential section of the gas attenuator to save space. The monitors are almost completely transparent due to the low pressure used for the rare gas in the vacuum chambers. An Si-based photodiode is also used to derive the pulse energy. Unlike gas-based detectors, the photodiode combined with a YAG screen is used to monitor both pulse energy and profile by interception technique.

## 3. Simulation results

### 3.1. Focus

In order to obtain an ideal focus spot in the experimental endstation, the wavefront should be well preserved in propagation. Therefore, the height errors of the mirrors in the beamline should be controlled at a reasonable level. According to the Maréchal criterion, the required height errors of the optics used in the beamline could be estimated with equation (1), 

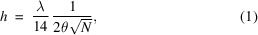

where *h* is the height error, λ is the wavelength, θ is the grazing angle and *N* is the number of optics. For the pink-beam branch, the beam passes through five mirrors. So, the height error for each mirror should be smaller than 0.76 nm RMS at 1000 eV and 1.22 nm RMS at 620 eV. The mirrors are obtained commercially from J-Tech Corporation. Table 2 ▸ shows the required and achieved parameters of the five mirrors used in the SASE beamline.

**Figure 2:**
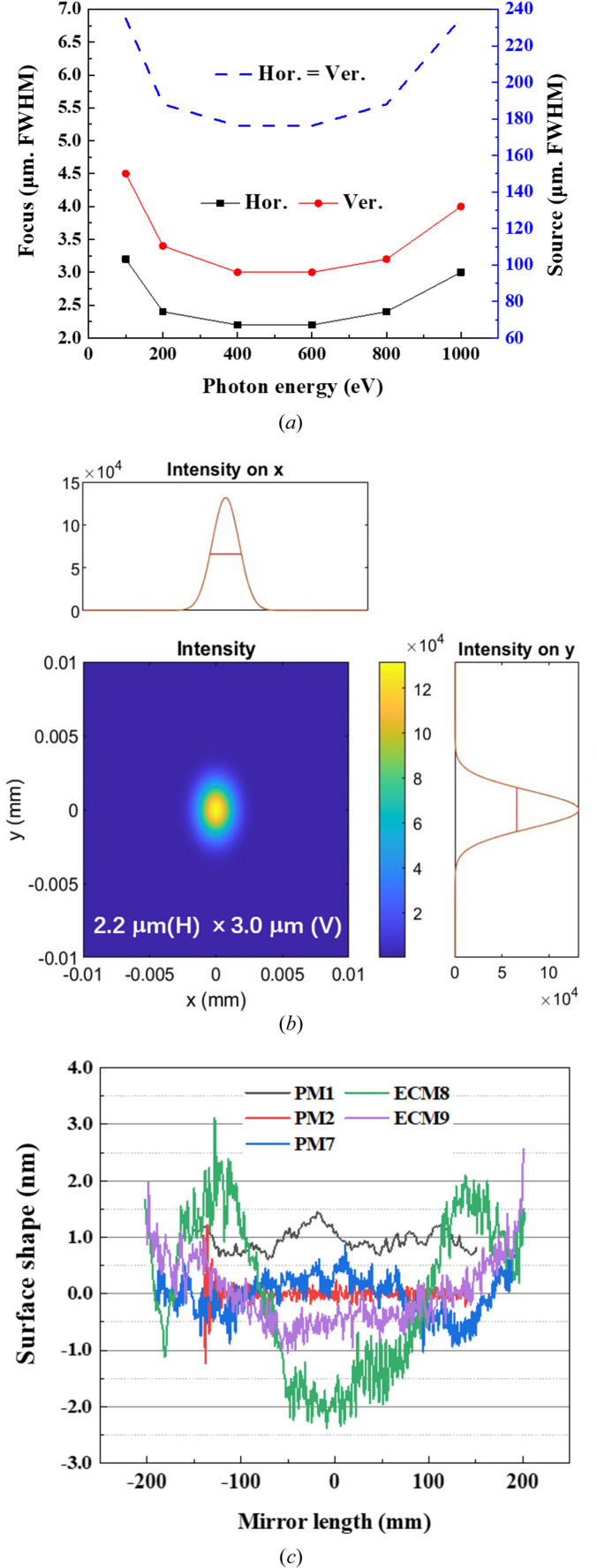
(*a*) Simulated focus size curves of the pink branch in the SASE beamline. (*b*) Simulated focus size of the pink branch in the SASE beamline at 600 eV. (*c*) The measured surface shape of the mirrors.

Simulation of the beam propagation is carried out using *MOI* code developed by SSRF (Meng *et al.*, 2015 ▸, 2017 ▸; Ren *et al.*, 2019 ▸, 2020 ▸). The model is based on statistical optics for numerical analysis of partially coherent X-rays. The simulated spot-size curve at the sample point of the pink branch is shown in Fig. 2 ▸(*a*), where the measured surface shape of the mirrors is adopted in the simulation. The focusing spot size at both ends of the energy range covered by the beamline is larger than that in the middle of the energy range, which is consistent with the trend of the source size [dashed line in Fig. 2 ▸(*a*)]. Figure 2 ▸(*b*) shows the simulated spot size at 600 eV. The ultra-smooth mirrors used in the beamline will contribute to a theoretical relatively ideal focusing spot. The measured surface shapes of the mirrors are shown in Fig. 2 ▸(*c*). Owing to the low repetition frequency, the focus would not be enlarged due the angular vibration of the mirrors especially for the single-pulse experiments, but the lateral position of the focus will be slightly affected.

### 3.2. Online spectrometer

**Figure 3:**
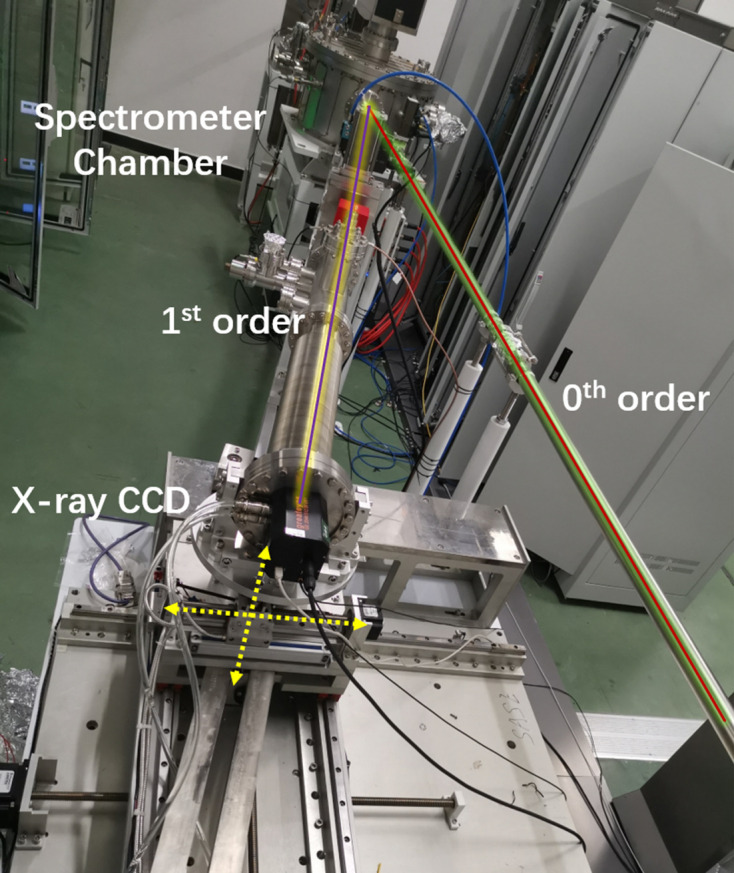
The online spectrometer of the SASE beamline.

In order to realize online measurements of FEL spectra, a VLS plane grating was ruled at the center of PM2. The incident FEL beam is dispersed by a grating of first order and recorded by an X-ray CCD, with the zeroth order being transported downstream, as shown in Fig. 3 ▸.

For a VLS grating, the line spacing *d* is a function of position *w* in the dispersive direction. The function can be expanded as a power series of *w*, namely,

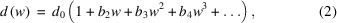

where *d*_0_ is the line spacing at the center of the grating, and *b*_2_, *b*_3_ and *b*_4_ are the space-variation parameters. The defocus term (*F*_20_) and the coma term (*F*_30_) in an optical path function can be eliminated by choosing an appropriate linear coefficient term *b*_2_ and quadratic term *b*_3_, respectively, according to

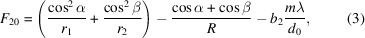




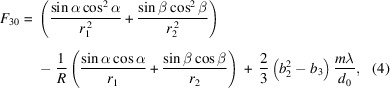

where *m* is the diffraction order, α is the incidence angle, β is the diffraction angle, *r*_1_ is the objective distance, *r*_2_ is the imaging distance and *R* is the curvature radius of the grating (for a plane grating, *R* → ∞). As an online spectrometer, the grating has to work at a fixed grazing incident angle to guarantee the normal transmission of the FEL beam. Therefore, the image distance of the grating has to vary with the change of photon energy to meet the focusing condition. Also, because of this, the defocus aberration could be eliminated over the whole energy range and contribute almost nothing to the energy-resolving power of the online spectrometer. The energy-resolving power is mainly determined by the following factors: source size, meridian slope error of the grating, aberration from the coma, detector and grating diffraction limit. High-order aberrations (smaller than *F*_30_) are small and negligible. Their contributions to the relative spectrum width, Δλ/λ_total_, where

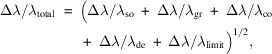

are as follows:

Source size:

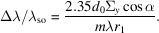

Meridian slopes errors of grating:

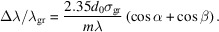

Detector:

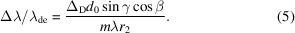

Aberration from coma:

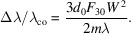

Diffraction limit:

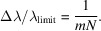

**Figure 4:**
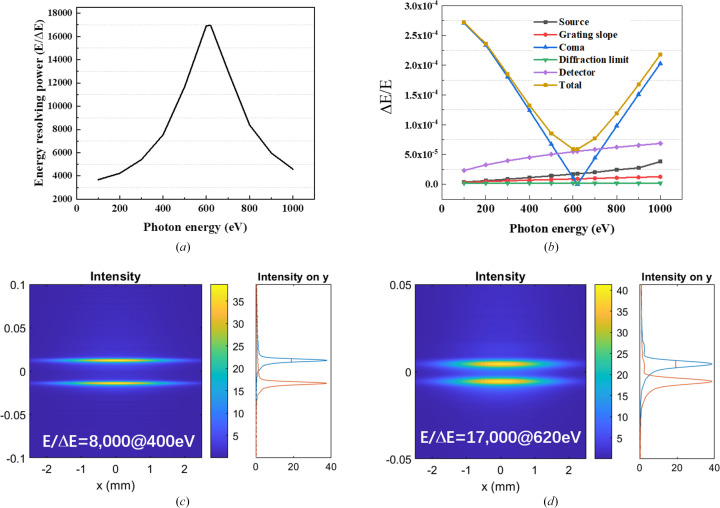
(*a*) Energy-resolving power of the online spectrometer. (*b*) The various contributions to the total energy resolution of the online spectrometer. (*c*) Ray-tracing result of the online spectrometer at 400 eV. (*d*) Ray-tracing result of the online spectrometer at 620 eV.

Here, Σ_*y*_ is the RMS value of the source size, σ_gr_ is the meridian RMS slope errors of the grating and the focusing mirror, *Δ*_D_ is the pixel size of the detector, γ is the grazing incidence angle on the CCD, *W* is the half-width of the ruled area of the grating and *N* is the number of coherently illuminated grating grooves. The theoretical energy-resolving power of the online spectrometer is shown in Fig. 4 ▸(*a*). The highest energy-resolving power is over 17000 at 620 eV, which is just the optimized energy for eliminating aberrations. This is mainly because the largest contribution to the energy-resolving power is from the coma aberration as shown in Fig. 4 ▸(*b*). The ray-tracing results also verify that the energy-resolving power could reach 8000 @ 400 eV and 17000 @ 620 eV, as shown in Figs. 4 ▸(*c*) and 4(*d*).

## 4. Initial commissioning results

### 4.1. Online spectrometer

**Figure 5:**
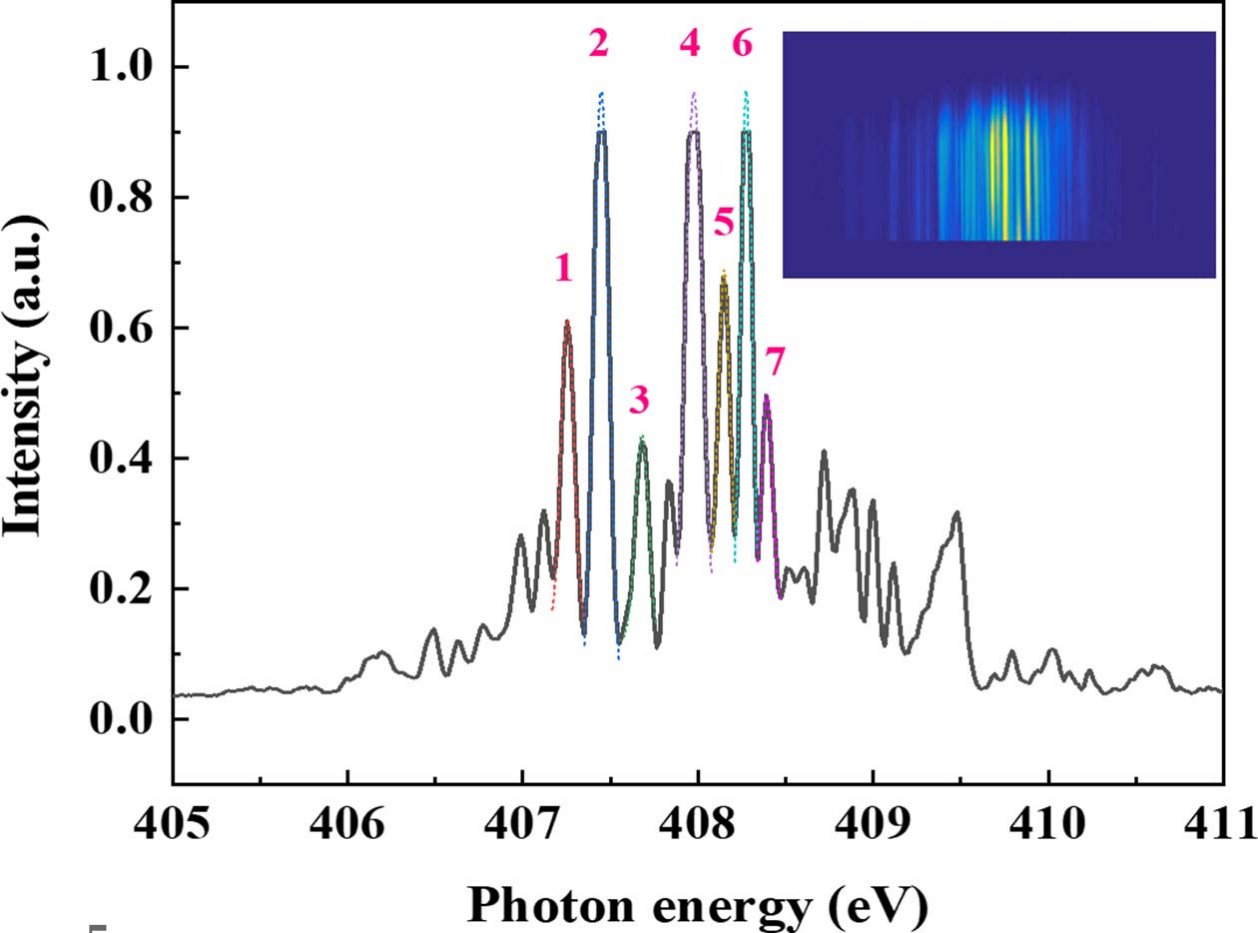
Single-shot spectra recorded by the online spectrometer. Numbering of the peaks refers to the peaks given in Table 3 ▸.

The online spectrometer is designed to allow the accurate measurement of the photon energy spectrum distribution over the whole energy range covered by the beamline while delivering radiation to the experimental endstation. The most important information directly obtained by using the online spectrometer is the energy distribution besides the central wavelength of the FEL pulses. Therefore, the online spectrometer is an important and essential diagnostic tool for both experimental scientists and machine scientists. The single-shot spectra could provide the spectral structures of the SASE FEL pulse by pulse. In the initial stage of beamline commissioning, a combination of visible-light CCD and YAG screen was used to record the spectra. Unfortunately, high-resolution spectra could not be obtained in this way. Afterwards, the detector of the spectrometer was replaced with an in-vacuum X-ray CCD camera. A randomly chosen SASE spike-like single-shot spectrum recorded by the X-ray CCD camera is presented in Fig. 5 ▸. Thanks to the high energy-resolving power of the online spectrometer, several Gaussian-shaped peaks can be observed in the spectra. The width of each peak can be obtained by Gaussian fitting, as listed in Table 3 ▸. A 60.5 meV energy resolution reveals that the energy-resolving power of the online spectrometer is over 6000 @ 400 eV.

**Figure 6:**
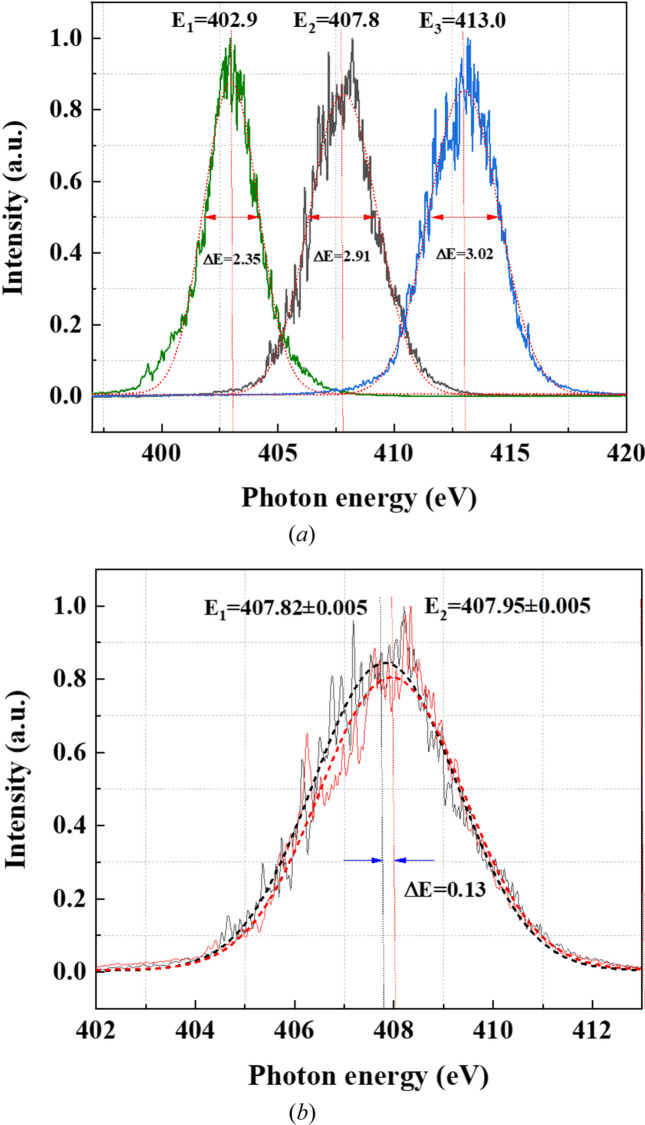
(*a*) Spectral distribution of the FEL source with different undulator gaps obtained by accumulating 100 single-shot spectra. (*b*) Spectral distribution of the FEL source measured when the undulator gap returned to its original value.

For the machine scientists, accurate spectral information assists in precise machine commissioning. A typical case is the calibration of the central wavelength of the FEL radiation and measurement of bandwidth. An experimental result is shown in Fig. 6 ▸. In this experiment the gap of the undulator is continuously adjusted twice with a step of 5.5 eV theoretically. One-hundred single-shot spectra were accumulated at each undulator gap to obtain the profile of the FEL radiation. Through Gaussian fitting of the measured source spectra profile, both the central wavelength and the bandwidth could be obtained. It can be seen from Fig. 6 ▸(*a*) that the actual change in the center wavelength is 4.9 eV and 5.2 eV and the bandwidth of the source at each undulator gap is 2.35 eV, 2.91 eV and 3.02 eV. Although there is a 0.6 eV and 0.3 eV difference between the actual change in the central wavelength and the theoretical expected value, these differences are smaller than the bandwidth of the source and still within an acceptable range. The measured multi-shot average source bandwidth is 0.6–0.7%, which is about three times the theoretical value of a single pulse of 0.2%. This is mainly caused by the energy jitter between pulses of the SASE radiation. A 130 meV difference of the central wavelength was also measured when the undulator gap returned to its original value as shown in Fig. 6 ▸(*b*).

### 4.2. Focal spot

In the first round of commissioning, the FEL pulses have been successfully transported and focused to the Coherent Scattering and Imaging (CSI) endstation. A bright X-ray focal spot of size 2.2 µm × 2.5 µm was achieved by using an edge-scan and silicon ablation imprint measurements at a photon energy of 520 eV (Tong *et al.*, 2022 ▸). The horizontal spot size is consistent with the theoretical simulation results, which reveals the high-quality transmission and focusing capabilities of the beamline, whereas the vertical spot size is even smaller than the results of theoretical predictions. A possible and reasonable reason for this is that the source size in the horizontal is not equal to that in the vertical while the source size adopted in the simulation is equal in both directions. Subsequently, the single-shot focal spot size was characterized using the coherent diffraction imaging reconstruction method (Gao *et al.*, 2023 ▸), which was consistent with that of the damage method and edge-scan method.

## 5. Conclusion

The Shanghai Soft X-ray Free-Electron Laser (SXFEL) is the first X-ray free-electron laser facility in China. The design of the SASE beamline, one of two beamlines in the Phase-1 construction, is presented in this paper. After the first round of commissioning the focal spot size in the pink branch is less than 3 µm in both the vertical and horizontal. The energy-resolving power of the online spectrometer is over 6000 @ 400 eV. The beamline and experimental station are now open to users. In the near future the commissioning of the more challenging mono-branch will start.

## Figures and Tables

**Table 1:** Basic parameters of the optics in the SASE beamline

Mirror	Location (m)	Type	Effective area (L × W) (mm)	Coating	Grazing incidence angle (°)
PM1	59	Plane	300 × 30	B_4_C	1.5
PM2	65	Plane	280 × 30	B_4_C	1.5
PM3	72.7	Plane	600 × 40	B_4_C	–
ECM4	73	Elliptical cylindrical	360 × 30	B_4_C	1.88
PG	73	Plane	190 × 30	B_4_C	–
ECM5	76	Elliptical cylindrical	400 × 40	B_4_C	1.2
EM 6	118.5	Ellipsoidal	490 × 20	B_4_C	1.5
PM7	117.5	Plane	380 × 30	B_4_C	1.5
ECM8	118	Elliptical cylindrical	410 × 30	B_4_C	1.5
ECM9	118.5	Elliptical cylindrical	410 × 30	B_4_C	1.5

**Table 2:** Required and achieved parameters of the optics used in the SASE beamline

		PM1	PM2	PM7	ECM8	ECM9
Height error (RMS) / peak-to-valley (nm)	Required	0.75 / 3				
	Achieved	0.24 / 1.42	0.17 / 2.2	0.34 / 2.39	1.4 / 5.8	0.74 / 4.09
Slope error (RMS) (nrad)	Required	200				
	Achieved	76	132	61	156	80

**Table 3:** Width of Gaussian-shaped peaks in a randomly chosen single-shot spectrum recorded by the online spectrometer

	Peak 1	Peak 2	Peak 3	Peak 4	Peak 5	Peak 6	Peak 7
Width (meV)	75.9 ± 5.5	90.9 ± 6.2	73.9 ± 4.4	99.0 ± 11.2	61.8 ± 2.9	65.4 ± 8.7	60.5 ± 2.5

## References

[bb1] Ackermann, W., Asova, G., Ayvazyan, V., Azima, A., Baboi, N., Bähr, J., Balandin, V., Beutner, B., Brandt, A., Bolzmann, A., Brinkmann, R., Brovko, O. I., Castellano, M., Castro, P., Catani, L., Chiadroni, E., Choroba, S., Cianchi, A., Costello, J. T., Cubaynes, D., Dardis, J., Decking, W., Delsim-Hashemi, H., Delserieys, A., Di Pirro, G., Dohlus, M., Düsterer, S., Eckhardt, A., Edwards, H. T., Faatz, B., Feldhaus, J., Flöttmann, K., Frisch, J., Fröhlich, L., Garvey, T., Gensch, U., Gerth, Ch., Görler, M., Golubeva, N., Grabosch, H.-J., Grecki, M., Grimm, O., Hacker, K., Hahn, U., Han, J. H., Honkavaara, K., Hott, T., Hüning, M., Ivanisenko, Y., Jaeschke, E., Jalmuzna, W., Jezynski, T., Kammering, R., Katalev, V., Kavanagh, K., Kennedy, E. T., Khodyachykh, S., Klose, K., Kocharyan, V., Körfer, M., Kollewe, M., Koprek, W., Korepanov, S., Kostin, D., Krassilnikov, M., Kube, G., Kuhlmann, M., Lewis, C. L. S., Lilje, L., Limberg, T., Lipka, D., Löhl, F., Luna, H., Luong, M., Martins, M., Meyer, M., Michelato, P., Miltchev, V., Möller, W. D., Monaco, L., Müller, W. F. O., Napieralski, O., Napoly, O., Nicolosi, P., Nölle, D., Nuñez, T., Oppelt, A., Pagani, C., Paparella, R., Pchalek, N., Pedregosa-Gutierrez, J., Petersen, B., Petrosyan, B., Petrosyan, G., Petrosyan, L., Pflüger, J., Plönjes, E., Poletto, L., Pozniak, K., Prat, E., Proch, D., Pucyk, P., Radcliffe, P., Redlin, H., Rehlich, K., Richter, M., Roehrs, M., Roensch, J., Romaniuk, R., Ross, M., Rossbach, J., Rybnikov, V., Sachwitz, M., Saldin, E. L., Sandner, W., Schlarb, H., Schmidt, B., Schmitz, M., Schmüser, P., Schneider, J. R., Schneidmiller, E. A., Schnepp, S., Schreiber, S., Seidel, M., Sertore, D., Shabunov, A. V., Simon, C., Simrock, S., Sombrowski, E., Sorokin, A. A., Spanknebel, P., Spesyvtsev, R., Staykov, L., Steffen, B., Stephan, F., Stulle, F., Thom, H., Tiedtke, K., Tischer, M., Toleikis, S., Treusch, R., Trines, D., Tsakov, I., Vogel, E., Weiland, T., Weise, H., Wellhöfer, M., Wendt, M., Will, I., Winter, A., Wittenburg, K., Wurth, W., Yeates, P., Yurkov, M. V., Zagorodnov, I. & Zapfe, K. (2007). *Nat. Photon.* **1**, 336–342.

[bb2] Allaria, E., Appio, R., Badano, L., Barletta, W. A., Bassanese, S., Biedron, S. G., Borga, A., Busetto, E., Castronovo, D., Cinquegrana, P., Cleva, S., Cocco, D., Cornacchia, M., Craievich, P., Cudin, I., D’Auria, G., Dal Forno, M., Danailov, M. B., De Monte, R., De Ninno, G., Delgiusto, P., Demidovich, A., Di Mitri, S., Diviacco, B., Fabris, A., Fabris, R., Fawley, W., Ferianis, M., Ferrari, E., Ferry, S., Froehlich, L., Furlan, P., Gaio, G., Gelmetti, F., Giannessi, L., Giannini, M., Gobessi, R., Ivanov, R., Karantzoulis, E., Lonza, M., Lutman, A., Mahieu, B., Milloch, M., Milton, S. V., Musardo, M., Nikolov, I., Noe, S., Parmigiani, F., Penco, G., Petronio, M., Pivetta, L., Predonzani, M., Rossi, F., Rumiz, L., Salom, A., Scafuri, C., Serpico, C., Sigalotti, P., Spampinati, S., Spezzani, C., Svandrlik, M., Svetina, C., Tazzari, S., Trovo, M., Umer, R., Vascotto, A., Veronese, M., Visintini, R., Zaccaria, M., Zangrando, D. & Zangrando, M. (2012). *Nat. Photon.* **6**, 699–704.

[bb3] Amann, J., Berg, W., Blank, V., Decker, F.-J., Ding, Y., Emma, P., Feng, Y., Frisch, J., Fritz, D., Hastings, J., Huang, Z., Krzywinski, J., Lindberg, R., Loos, H., Lutman, A., Nuhn, H.-D., Ratner, D., Rzepiela, J., Shu, D., Shvyd’ko, Yu., Spampinati, S., Stoupin, S., Terentyev, S., Trakhtenberg, E., Walz, D., Welch, J., Wu, J., Zholents, A. & Zhu, D. (2012). *Nat. Photon.* **6**, 693–698.

[bb4] Beaud, P., Caviezel, A., Mariager, S. O., Rettig, L., Ingold, G., Dornes, C., Huang, S.-W., Johnson, J. A., Radovic, M., Huber, T., Kubacka, T., Ferrer, A., Lemke, H. T., Chollet, M., Zhu, D., Glownia, J. M., Sikorski, M., Robert, A., Wadati, H., Nakamura, M., Kawasaki, M., Tokura, Y., Johnson, S. L. & Staub, U. (2014). *Nat. Mater.* **13**, 923–927. 10.1038/nmat4046 25087068

[bb5] Decking, W., Abeghyan, S., Abramian, P., Abramsky, A., Aguirre, A., Albrecht, C., Alou, P., Altarelli, M., Altmann, P., Amyan, K., Anashin, V., Apostolov, E., Appel, K., Auguste, D., Ayvazyan, V., Baark, S., Babies, F., Baboi, N., Bak, P., Balandin, V., Baldinger, R., Baranasic, B., Barbanotti, S., Belikov, O., Belokurov, V., Belova, L., Belyakov, V., Berry, S., Bertucci, M., Beutner, B., Block, A., Blöcher, M., Böckmann, T., Bohm, C., Böhnert, M., Bondar, V., Bondarchuk, E., Bonezzi, M., Borowiec, P., Bösch, C., Bösenberg, U., Bosotti, A., Böspflug, R., Bousonville, M., Boyd, E., Bozhko, Y., Brand, A., Branlard, J., Briechle, S., Brinker, F., Brinker, S., Brinkmann, R., Brockhauser, S., Brovko, O., Brück, H., Brüdgam, A., Butkowski, L., Büttner, T., Calero, J., Castro-Carballo, E., Cattalanotto, G., Charrier, J., Chen, J., Cherepenko, A., Cheskidov, V., Chiodini, M., Chong, A., Choroba, S., Chorowski, M., Churanov, D., Cichalewski, W., Clausen, M., Clement, W., Cloué, C., Cobos, J. A., Coppola, N., Cunis, S., Czuba, K., Czwalinna, M., D’Almagne, B., Dammann, J., Danared, H., de Zubiaurre Wagner, A., Delfs, A., Delfs, T., Dietrich, F., Dietrich, T., Dohlus, M., Dommach, M., Donat, A., Dong, X., Doynikov, N., Dressel, M., Duda, M., Duda, P., Eckoldt, H., Ehsan, W., Eidam, J., Eints, F., Engling, C., Englisch, U., Ermakov, A., Escherich, K., Eschke, J., Saldin, E., Faesing, M., Fallou, A., Felber, M., Fenner, M., Fernandes, B., Fernández, J. M., Feuker, S., Filippakopoulos, K., Floettmann, K., Fogel, V., Fontaine, M., Francés, A., Martin, I. F., Freund, W., Freyermuth, T., Friedland, M., Fröhlich, L., Fusetti, M., Fydrych, J., Gallas, A., García, O., Garcia-Tabares, L., Geloni, G., Gerasimova, N., Gerth, C., Geßler, P., Gharibyan, V., Gloor, M., Głowinkowski, J., Goessel, A., Gołębiewski, Z., Golubeva, N., Grabowski, W., Graeff, W., Grebentsov, A., Grecki, M., Grevsmuehl, T., Gross, M., Grosse-Wortmann, U., Grünert, J., Grunewald, S., Grzegory, P., Feng, G., Guler, H., Gusev, G., Gutierrez, J. L., Hagge, L., Hamberg, M., Hanneken, R., Harms, E., Hartl, I., Hauberg, A., Hauf, S., Hauschildt, J., Hauser, J., Havlicek, J., Hedqvist, A., Heidbrook, N., Hellberg, F., Henning, D., Hensler, O., Hermann, T., Hidvégi, A., Hierholzer, M., Hintz, H., Hoffmann, F., Hoffmann, M., Hoffmann, M., Holler, Y., Hüning, M., Ignatenko, A., Ilchen, M., Iluk, A., Iversen, J., Iversen, J., Izquierdo, M., Jachmann, L., Jardon, N., Jastrow, U., Jensch, K., Jensen, J., Jeżabek, M., Jidda, M., Jin, H., Johansson, N., Jonas, R., Kaabi, W., Kaefer, D., Kammering, R., Kapitza, H., Karabekyan, S., Karstensen, S., Kasprzak, K., Katalev, V., Keese, D., Keil, B., Kholopov, M., Killenberger, M., Kitaev, B., Klimchenko, Y., Klos, R., Knebel, L., Koch, A., Koepke, M., Köhler, S., Köhler, W., Kohlstrunk, N., Konopkova, Z., Konstantinov, A., Kook, W., Koprek, W., Körfer, M., Korth, O., Kosarev, A., Kosiński, K., Kostin, D., Kot, Y., Kotarba, A., Kozak, T., Kozak, V., Kramert, R., Krasilnikov, M., Krasnov, A., Krause, B., Kravchuk, L., Krebs, O., Kretschmer, R., Kreutzkamp, J., Kröplin, O., Krzysik, K., Kube, G., Kuehn, H., Kujala, N., Kulikov, V., Kuzminych, V., La Civita, D., Lacroix, M., Lamb, T., Lancetov, A., Larsson, M., Le Pinvidic, D., Lederer, S., Lensch, T., Lenz, D., Leuschner, A., Levenhagen, F., Li, Y., Liebing, J., Lilje, L., Limberg, T., Lipka, D., List, B., Liu, J., Liu, S., Lorbeer, B., Lorkiewicz, J., Lu, H. H., Ludwig, F., Machau, K., Maciocha, W., Madec, C., Magueur, C., Maiano, C., Maksimova, I., Malcher, K., Maltezopoulos, T., Mamoshkina, E., Manschwetus, B., Marcellini, F., Marinkovic, G., Martinez, T., Martirosyan, H., Maschmann, W., Maslov, M., Matheisen, A., Mavric, U., Meißner, J., Meissner, K., Messerschmidt, M., Meyners, N., Michalski, G., Michelato, P., Mildner, N., Moe, M., Moglia, F., Mohr, C., Mohr, S., Möller, W., Mommerz, M., Monaco, L., Montiel, C., Moretti, M., Morozov, I., Morozov, P., Mross, D., Mueller, J., Müller, C., Müller, J., Müller, K., Munilla, J., Münnich, A., Muratov, V., Napoly, O., Näser, B., Nefedov, N., Neumann, R., Neumann, R., Ngada, N., Noelle, D., Obier, F., Okunev, I., Oliver, J. A., Omet, M., Oppelt, A., Ottmar, A., Oublaid, M., Pagani, C., Paparella, R., Paramonov, V., Peitzmann, C., Penning, J., Perus, A., Peters, F., Petersen, B., Petrov, A., Petrov, I., Pfeiffer, S., Pflüger, J., Philipp, S., Pienaud, Y., Pierini, P., Pivovarov, S., Planas, M., Pławski, E., Pohl, M., Polinski, J., Popov, V., Prat, S., Prenting, J., Priebe, G., Pryschelski, H., Przygoda, K., Pyata, E., Racky, B., Rathjen, A., Ratuschni, W., Regnaud-Campderros, S., Rehlich, K., Reschke, D., Robson, C., Roever, J., Roggli, M., Rothenburg, J., Rusiński, E., Rybaniec, R., Sahling, H., Salmani, M., Samoylova, L., Sanzone, D., Saretzki, F., Sawlanski, O., Schaffran, J., Schlarb, H., Schlösser, M., Schlott, V., Schmidt, C., Schmidt-Foehre, F., Schmitz, M., Schmökel, M., Schnautz, T., Schneidmiller, E., Scholz, M., Schöneburg, B., Schultze, J., Schulz, C., Schwarz, A., Sekutowicz, J., Sellmann, D., Semenov, E., Serkez, S., Sertore, D., Shehzad, N., Shemarykin, P., Shi, L., Sienkiewicz, M., Sikora, D., Sikorski, M., Silenzi, A., Simon, C., Singer, W., Singer, X., Sinn, H., Sinram, K., Skvorodnev, N., Smirnow, P., Sommer, T., Sorokin, A., Stadler, M., Steckel, M., Steffen, B., Steinhau-Kühl, N., Stephan, F., Stodulski, M., Stolper, M., Sulimov, A., Susen, R., Świerblewski, J., Sydlo, C., Syresin, E., Sytchev, V., Szuba, J., Tesch, N., Thie, J., Thiebault, A., Tiedtke, K., Tischhauser, D., Tolkiehn, J., Tomin, S., Tonisch, F., Toral, F., Torbin, I., Trapp, A., Treyer, D., Trowitzsch, G., Trublet, T., Tschentscher, T., Ullrich, F., Vannoni, M., Varela, P., Varghese, G., Vashchenko, G., Vasic, M., Vazquez-Velez, C., Verguet, A., Vilcins-Czvitkovits, S., Villanueva, R., Visentin, B., Viti, M., Vogel, E., Volobuev, E., Wagner, R., Walker, N., Wamsat, T., Weddig, H., Weichert, G., Weise, H., Wenndorf, R., Werner, M., Wichmann, R., Wiebers, C., Wiencek, M., Wilksen, T., Will, I., Winkelmann, L., Winkowski, M., Wittenburg, K., Witzig, A., Wlk, P., Wohlenberg, T., Wojciechowski, M., Wolff-Fabris, F., Wrochna, G., Wrona, K., Yakopov, M., Yang, B., Yang, F., Yurkov, M., Zagorodnov, I., Zalden, P., Zavadtsev, A., Zavadtsev, D., Zhirnov, A., Zhukov, A., Ziemann, V., Zolotov, A., Zolotukhina, N., Zummack, F. & Zybin, D. (2020). *Nat. Photon.* **14**, 391–397.

[bb7] Emma, P., Akre, R., Arthur, J., Bionta, R., Bostedt, C., Bozek, J., Brachmann, A., Bucksbaum, P., Coffee, R., Decker, F.-J., Ding, Y., Dowell, D., Edstrom, S., Fisher, A., Frisch, J., Gilevich, S., Hastings, J., Hays, G., Hering, Ph., Huang, Z., Iverson, R., Loos, H., Messerschmidt, M., Miahnahri, A., Moeller, S., Nuhn, H.-D., Pile, G., Ratner, D., Rzepiela, J., Schultz, D., Smith, T., Stefan, P., Tompkins, H., Turner, J., Welch, J., White, W., Wu, J., Yocky, G. & Galayda, J. (2010). *Nat. Photon.* **4**, 641–647.

[bb25] Gao, Z., Fan, J., Tong, Y., Zhang, J., He, B., Nie, Y., Luan, H., Lu, D., Zhang, D., Yuan, X., Wang, Y., Liu, Z. & Jiang, H. (2023). *J. Synchrotron Rad.* **30**, 505–513. 10.1107/S1600577523000887 PMC10161889 36947163

[bb18] Ishikawa, T., Aoyagi, H., Asaka, T., Asano, Y., Azumi, N., Bizen, T., Ego, H., Fukami, K., Fukui, T., Furukawa, Y., Goto, S., Hanaki, H., Hara, T., Hasegawa, T., Hatsui, T., Higashiya, A., Hirono, T., Hosoda, N., Ishii, M., Inagaki, T., Inubushi, Y., Itoga, T., Joti, Y., Kago, M., Kameshima, T., Kimura, H., Kirihara, Y., Kiyomichi, A., Kobayashi, T., Kondo, C., Kudo, T., Maesaka, H., Maréchal, X. M., Masuda, T., Matsubara, S., Matsumoto, T., Matsushita, T., Matsui, S., Nagasono, M., Nariyama, N., Ohashi, H., Ohata, T., Ohshima, T., Ono, S., Otake, Y., Saji, C., Sakurai, T., Sato, T., Sawada, K., Seike, T., Shirasawa, K., Sugimoto, T., Suzuki, S., Takahashi, S., Takebe, H., Takeshita, K., Tamasaku, K., Tanaka, H., Tanaka, R., Tanaka, T., Togashi, T., Togawa, K., Tokuhisa, A., Tomizawa, H., Tono, K., Wu, S., Yabashi, M., Yamaga, M., Yamashita, A., Yanagida, K., Zhang, C., Shintake, T., Kitamura, H. & Kumagai, N. (2012). *Nat. Photon.* **6**, 540–544.

[bb8] Kang, H., Min, C., Heo, H., Kim, C., Yang, H., Kim, G., Nam, I., Baek, S. Y., Choi, H., Mun, G., Park, B. R., Suh, Y. J., Shin, D. C., Hu, J., Hong, J., Jung, S., Kim, S., Kim, K., Na, D., Park, S. S., Park, Y. J., Han, J., Jung, Y. G., Jeong, S. H., Lee, H. G., Lee, S., Lee, S., Lee, W., Oh, B., Suh, H. S., Parc, Y. W., Park, S., Kim, M. H., Jung, N., Kim, Y., Lee, M., Lee, B., Sung, C., Mok, I., Yang, J., Lee, C., Shin, H., Kim, J. H., Kim, Y., Lee, J. H., Park, S., Kim, J., Park, J., Eom, I., Rah, S., Kim, S., Nam, K. H., Park, J., Park, J., Kim, S., Kwon, S., Park, S. H., Kim, K. S., Hyun, H., Kim, S. N., Kim, S., Hwang, S., Kim, M. J., Lim, C., Yu, C., Kim, B., Kang, T., Kim, K., Kim, S., Lee, H., Lee, H., Park, K., Koo, T., Kim, D. & Ko, I. S. (2017). *Nat. Photon.* **11**, 708–713.

[bb9] Liu, B., Feng, C., Gu, D., Gao, F., Deng, H., Zhang, M., Sun, S., Chen, S., Zhang, W., Fang, W., Wang, Z., Zhou, Q., Leng, Y., Gu, M., Yin, L., Gu, Q., Fang, G., Wang, D. & Zhao, Z. (2022). *Appl. Sci.* **12**, 176.

[bb22] Liu, W., Wacker, D., Gati, C., Han, G. W., James, D., Wang, D., Nelson, G., Weierstall, U., Katritch, V., Barty, A., Zatsepin, N. A., Li, D., Messerschmidt, M., Boutet, S., Williams, G. J., Koglin, J. E., Seibert, M. M., Wang, C., Shah, S. T. A., Basu, S., Fromme, R., Kupitz, C., Rendek, K. N., Grotjohann, I., Fromme, P., Kirian, R. A., Beyerlein, K. R., White, T. A., Chapman, H. N., Caffrey, M., Spence, J. C. H., Stevens, R. C. & Cherezov, V. (2013). *Science*, **342**, 1521–1524. 10.1126/science.1244142 PMC3902108 24357322

[bb10] Mankowsky, R., Subedi, A., Först, M., Mariager, S. O., Chollet, M., Lemke, H. T., Robinson, J. S., Glownia, J. M., Minitti, M. P., Frano, A., Fechner, M., Spaldin, N. A., Loew, T., Keimer, B., Georges, A. & Cavalleri, A. (2014). *Nature*, **516**, 71–73. 10.1038/nature13875 25471882

[bb11] McNeil, B. W. J. & Thompson, N. R. (2010). *Nat. Photon.* **4**, 814–821.

[bb12] Meng, X., Shi, X., Wang, Y., Reininger, R., Assoufid, L. & Tai, R. (2017). *J. Synchrotron Rad.* **24**, 954–962. 10.1107/S1600577517010281 28862617

[bb13] Meng, X., Xue, C., Yu, H., Wang, Y., Wu, Y. & Tai, R. (2015). *Opt. Express*, **23**, 29675–29686. 10.1364/OE.23.029675 26698449

[bb6] Prat, E., Abela, R., Aiba, M., Alarcon, A., Alex, J., Arbelo, Y., Arrell, C., Arsov, V., Bacellar, C., Beard, C., Beaud, P., Bettoni, S., Biffiger, R., Bopp, M., Braun, H., Calvi, M., Cassar, A., Celcer, T., Chergui, M., Chevtsov, P., Cirelli, C., Citterio, A., Craievich, P., Divall, M. C., Dax, A., Dehler, M., Deng, Y., Dietrich, A., Dijkstal, P., Dinapoli, R., Dordevic, S., Ebner, S., Engeler, D., Erny, C., Esposito, V., Ferrari, E., Flechsig, U., Follath, R., Frei, F., Ganter, R., Garvey, T., Geng, Z., Gobbo, A., Gough, C., Hauff, A., Hauri, C. P., Hiller, N., Hunziker, S., Huppert, M., Ingold, G., Ischebeck, R., Janousch, M., Johnson, P. J. M., Johnson, S. L., Juranić, P., Jurcevic, M., Kaiser, M., Kalt, R., Keil, B., Kiselev, D., Kittel, C., Knopp, G., Koprek, W., Laznovsky, M., Lemke, H. T., Sancho, D. L., Löhl, F., Malyzhenkov, A., Mancini, G. F., Mankowsky, R., Marcellini, F., Marinkovic, G., Martiel, I., Märki, F., Milne, C. J., Mozzanica, A., Nass, K., Orlandi, G. L., Loch, C. O., Paraliev, M., Patterson, B., Patthey, L., Pedrini, B., Pedrozzi, M., Pradervand, C., Radi, P., Raguin, J., Redford, S., Rehanek, J., Reiche, S., Rivkin, L., Romann, A., Sala, L., Sander, M., Schietinger, T., Schilcher, T., Schlott, V., Schmidt, T., Seidel, M., Stadler, M., Stingelin, L., Svetina, C., Treyer, D. M., Trisorio, A., Vicario, C., Voulot, D., Wrulich, A., Zerdane, S. & Zimoch, E. (2020). *Nat. Photon.* **14**, 748–754.

[bb14] Ren, J., Meng, X., Wang, Y., Cao, J., Li, J. & Tai, R. (2020). *J. Synchrotron Rad.* **27**, 1485–1493. 10.1107/S1600577520010565 33147173

[bb15] Ren, J., Wang, Y., Meng, X., Shi, X., Assoufid, L. & Tai, R. (2019). *J. Synchrotron Rad.* **26**, 1198–1207. 10.1107/S1600577519005253 31274444

[bb16] Rohringer, N. & Santra, R. (2007). *Phys. Rev. A*, **76**, 033416.

[bb17] Saldin, E. L., Schneidmiller, E. A. & Yurkov, M. V. (1995). *Phys. Rep.* **260**, 187–327.

[bb19] Tiedtke, K., Feldhaus, J., Gerth, Ch., Hahn, U., Jastrow, U., Ploenjes, E., Steeg, B. & Treusch, R. (2004). *AIP Conf. Proc.* **705**, 588–592.

[bb20] Togashi, T., Takahashi, E. J., Midorikawa, K., Aoyama, M., Yamakawa, K., Sato, T., Iwasaki, A., Owada, S., Yamanouchi, K., Hara, T., Matsubara, S., Ohshima, T., Otake, Y., Tamasaku, K., Tanaka, H., Tanaka, T., Tomizawa, H., Watanabe, T., Yabashi, M. & Ishikawa, T. (2013). *Radiat. Phys. Chem.* **93**, 25–32.

[bb21] Tong, Y., Fan, J., Nie, Y., Guo, Z., Gao, Z., Yuan, X., He, B., Chen, J., Zhang, D., Luan, H., Zhang, J., Lu, D., Xie, M., Cheng, P., Feng, C., Liu, T., Deng, H., Liu, B., Liu, Z. & Jiang, H. (2022). *Front. Phys.* **10**, 977957.

[bb23] Wernet, Ph., Kunnus, K., Josefsson, I., Rajkovic, I., Quevedo, W., Beye, M., Schreck, S., Grübel, S., Scholz, M., Nordlund, D., Zhang, W., Hartsock, R. W., Schlotter, W. F., Turner, J. J., Kennedy, B., Hennies, F., de Groot, F. M. F., Gaffney, K. J., Techert, S., Odelius, M. & Föhlisch, A. (2015). *Nature*, **520**, 78–81. 10.1038/nature14296 25832405

[bb24] Zhao, Z., Wang, D., Gu, Q., Yin, L., Gu, M., Leng, Y. & Liu, B. (2017). *Appl. Sci.* **7**, 607.

